# A review on functional lung avoidance radiotherapy plan for lung cancer

**DOI:** 10.3389/fonc.2024.1429837

**Published:** 2024-12-05

**Authors:** Jinhui Yu, Xiaofeng Tang, Yifan Lei, Zhe Zhang, Bo Li, Han Bai, Lan Li

**Affiliations:** ^1^ The Third Affiliated Hospital of Kunming Medical University, Kunming Medical University, Kunming, Yunnan, China; ^2^ Department of Radiation Oncology, The Third Affiliated Hospital of Kunming Medical University, Yunnan Tumor Hospital, Kunming, Yunnan, China; ^3^ Department of Physics and Astronomy, Yunnan University, Kunming, Yunnan, China

**Keywords:** radiotherapy, lung function imaging, lung functional image-based radiotherapy, radiation-induced lung injury, clinical benefit

## Abstract

Lung cancer is the most common malignant tumor in China. Its incidence and mortality rate increase year by year. In the synthesis treatment of lung cancer, radiotherapy (RT) plays a vital role, and radiation-induced lung injury(RILI) has become the major limiting factor in prescription dose escalation. Conventional RT is designed to minimize radiation exposure to healthy lungs without considering the inhomogeneity of lung function, which is significantly non-uniform in most patients. In accordance with the functional and structural heterogeneity of lung tissue, functional lung avoidance RT (FLART) can reduce radiation exposure to functional lung (FL), thus reducing RILI. Meanwhile, a dose-function histogram (DFH) was proposed to describe the dose parameters of the optimized image-guided RT plan. This paper reviews lung function imaging for lung cancer RT plans. It also reviews the clinical applications of function-guided RT plans and their current problems and research directions to provide better guidance for clinical selection.

## 1 Introduction

As the most prevalent malignancy worldwide, lung cancer is the main cause of death. Up to 77% of patients require RT during treatment (1). In some patients, RILI is significant. RILI caused by excessive radiation doses to healthy lungs is a limiting factor regarding radiation doses, treatment appropriateness, quality of life following treatment, and the appropriateness of newly introduced adjuvant immunotherapy (2). Modern advanced RT technologies, including intensity-modulated radiation therapy (IMRT) (3), volumetric modulated arc therapy (VMAT) (4), stereotactic body radiation therapy (SBRT) (5), and proton radiation therapy (PRT) (6), can meet conformal requirements of target volume and dose limitation requirements of normal tissues. Modern RT techniques aim to minimize the radiation dose to healthy lungs without considering the regional distribution of the lung function. Many lung cancer patients have significant regional differences in lung function due to smoking and chronic lung complications (7), and different parts and functional states respond differently to radiation; Studies have shown that the better the functional status, the more sensitive it is to radiation (8, 9). Functional images obtained with imaging techniques, such as magnetic resonance imaging (MRI) (10), computed tomography (CT) (11), and nuclear medicine (12), provide physiological information that can be used in RT planning. Studies have shown that the correlation between radiation pneumonitis (RP) and features calculated from functional images (13, 14) is better than that of conventional dose and volume histogram (DVH) features (15, 16). The traditional DVH algorithm regards the whole structure as a functionally uniform whole, ignoring functional and structural heterogeneity (15). DFH, as the evaluation parameter of functionally guided RT planning, groups the number of each sample point associated with the physiological or functional status. This is done using the dose and ventilation value in each pixel calculated to obtain DFH (13). It is possible to evaluate FLART plans using DFH parameters such as the mean lung function-weighted dose (f-MLD) and function-weighted V_x_ (fV_5_, fV_10_, and fV_20_) (16–19). DFH was calculated from perfusion (Q) and ventilation (V) values and the dose for each voxel (20–23), where f-MLD is obtained by weighting each dose voxel based on its V or Q value, and fV_20_ is defined as the percentage of total V or Q contained in the volume receiving a 20 Gy dose (24). As reported by Yamamoto et al. (25, 26), functional image-guided RT plans aim to selectively avoid irradiation of the functional regions of the lung. This reduces the probability of lung toxicity after RT while meeting standard dose limits for critical organs. This review focuses on functional imaging techniques commonly used in the literature. It also focuses on the research progress based on functional image-guided RT plans and some current problems faced. Additionally, some novel RT techniques that can be combined with adaptive radiation therapy (ART) are reviewed for FLART plans.

## 2 Nuclear medicine image-guided FLART

Lung functional measurements and mapping are used to evaluate lung cancer RT plans. Various lung functional imaging have been studied for lung cancer RT, such as nuclear medicine imaging, including single-photon emission computed tomography (SPECT) (2, 27, 28) and positron emission tomography/computed tomography (PET/CT) (17, 29). SPECT and PET/CT can provide lung Q and V information and perform three-dimensional (3D) imaging. Studies recommend the use of lung functional imaging for guiding lung cancer RT (2, 17, 21, 27–29). This is aimed at minimizing the radiation dose in functionally normal lung regions while maintaining the therapeutic dose at the planning target volume (PTV).

### 2.1 SPECT

SPECT is a recognized functional imaging modality for diagnosing and monitoring lung diseases (30), and SPECT-based Q imaging was the earliest reported form of lung functional imaging (22). SPECT can be used for lung Q- and V-imaging. Radioactive tracers commonly used in SPECT V imaging include gas (^133^Xe and ^81m^Kr), aerosol (^99m^Tc-DTPA), and solid (^99m^Tc-Technegas) tracers. Radioactive Q tracers commonly employ technetium-99m-labeled macroaggregated albumin (^99m^Tc-MAA) (31– 32).

#### 2.1.1 Lung functional imaging based on SPECT

The imaging quality of V was influenced by the size of the tracer. Patients with potential airway diseases have a higher risk of central and focal peripheral radioactive tracer deposition. As a result, SPECT-based functional imaging most commonly uses Q imaging (33). According to Hoover et al. (21), the area under the curve (AUC) values of DFH parameters weighted by Q were consistently higher than those weighted by V and their corresponding standard DVH parameters. Radioactive Q tracers enter the human body through the veins, circulate with the blood to the vascular bed tissue in the lungs through heart Q, and reflect the Q status of pulmonary blood flow. If a capillary embolism occurred in the lungs, the imaging agent was retained. Lung Q imaging can measure blood circulation in the lungs and is commonly used in clinics to display regional functional information (2). This viewpoint was also supported by previous animal experiments (34). Furthermore, several surgical studies in human patients have shown a reasonable correlation between the percentage of Q lungs resected and the percentage of lung function decline (35, 36). Patients receiving lung RT usually undergo CT and SPECT at the treatment site, and the structure to be evaluated is determined by continuously drawing contours on the CT scan images (22). The 3D images of Q were segmented based on pixel values, with higher pixel values corresponding to higher Q areas in the lungs, thus representing higher functionality (37).

#### 2.1.2 FLART plans based on SPECT Lung functional imaging

SPECT uses radioactively labeled tracers to image lung circulation, where the Q region is equivalent to a normal functioning lung (2). In the evaluation of lung function, Q has shown predictive potential for RILI in lung cancer and other cancers involving lung RT (17, 38–40). An FL contour was created for each patient from the SPECT signal, and these contours were transferred from SPECT to the planning CT using image registration and cropped to depict the total lung volume (2). SPECT images can be integrated into numerical optimization because the count rate is linearly correlated with Q, and the regional function approximates linearly with regional Q (41). Based on published literature, the most commonly used threshold in SPECT functional zoning was 30% (42–46), followed by 50% (17, 47, 48) and 70% (17, 48, 49). Dhami et al. (50) found that 70% of the maximum Q threshold in SPECT imaging was most correlated with RP clinical endpoints. Meanwhile, Hoover et al. (21) weighted the areas exceeding 30% of the maximum Q count in SPECT with FL using dynamic blood flow images. The remaining lung tissue, which accounted for less than 30% of function, was called the non-functional lung (NFL). In the study by Shioyama et al. (47), SPECT adopts functional plans with thresholds of 50% and 90%. Compared to the anatomical plans, the median decrease in f-MLD was 2.2 Gy (17.6-14.5) and 4.2 Gy (16.0-111.8), respectively. Similar results have also been shown in other studies (43, 44, 49). It was possible to correlate pulmonary toxicity with lung function subunit quantities that exceed a certain threshold dose by scaling the volume of individual voxels according to the local SPECT intensity (21). The fusion of the Q image with the original treatment plan generated a baseline DFH, which displayed the relationship between radiation dose and functional region volume. Furthermore, treatment plan optimization could be attained by reducing the average Q-weighted lung dose derived from the histogram of the blood-flow-driven Q function (51). Additionally, Christian et al. (52) proposed minimizing the dose to the FL volume outlined on the Q-map as a planning objective.

#### 2.1.3 Clinical benefits from FLART based on SPECT lung functional imaging

Despite these differences, all studies indicated that FLART could improve lung function protection, at least in a specific subset of patients (e.g., patients with large Q defects) (53). This improvement was usually reflected in reducing certain DVH parameters of the total lung (16, 24, 54) or certain DVH parameters of the FL (15, 16, 25). Earlier studies have shown that Q-weighted optimization-guided RT plans reduce RILI in patients with large Q defects in non-small cell lung cancer (NSCLC) (47, 51), but the reduction is less significant in patients with small Q defects. Studies by Farr et al. (55) demonstrated that f-MLD and MLD predicted the AUC of G^2+^RP at 0.812 and 0.716, respectively, and fV_20_ and V_20_ predicted the AUC of G^2+^RP at 0.792 and 0.716, respectively. Another study has yielded similar results (21).

### 2.2 PET/CT

With the rapid development of medical imaging technology, the application of PET/CT functional imaging in tumor RT is becoming increasingly widespread. With PET/CT, images are generated that integrate the anatomical structure and functional metabolism of patients under the same conditions, thus providing a more accurate imaging basis for treatment (56, 57). Compared to SPECT, PET has higher resolution and sensitivity, can provide more physiological and functional information, and lowers radioactive drug radiation doses (58–60). It can also be fused with imaging technologies such as CT or MRI to provide more comprehensive diagnostic information and can even be combined with four-dimensional X-ray CT (4DCT) to obtain 4D V and Q information (61–64).

#### 2.2.1 Lung functional imaging based on PET/CT

PET imaging uses the decay mechanism of radioactive isotopes. During the decay process, radioactive isotopes release positrons that undergo annihilation when they encounter electrons, resulting in the production of two gamma rays. These rays are emitted at an 180-degree angle, and PET scanners detect the relative positions of these rays to reconstruct an image of the distribution of radioactive tracers inside the body. Previous studies have mainly used PET tracers, such as ^15^O, ^13^N, and ^11^C for pulmonary physiology research. However, due to their short half-lives, they are gradually being replaced by galligas and gallium-68 macroaggregated albumin (^68^Ga-MAA) for lung V/Q studies (64–67). PET V imaging was performed first, followed by lung Q imaging, all without moving the patient as in the SPECT V/Q program using aerosols. ^68^Ga was first used for PET Q imaging in 1976 by Chesler et al. (68), who injected radiolabeled albumin microspheres containing ^68^Ga into dogs to obtain 3D reconstructed images. In 2010, Kotzerke et al. (69) first described the use of galligas for V-PET imaging. However, owing to the influence of respiratory motion, there may be significant misalignment between PET and CT. PET/CT images can be improved by using the respiratory gate technique (4D PET/CT), which reduces artifacts caused by respiratory motion during imaging (64). In a study by Siva et al. (44), the operational procedure for performing V/Q imaging using 4D PET/CT was described. Patients first inhaled galligas and underwent V imaging with a respiratory tracking system used during the scan. Patients were instructed to breathe freely while simultaneously undergoing a low-dose 4DCT chest acquisition. The scan ranges for both 4D PET and 4DCT include the entire lung field. After the lung V-PET scan, approximately 40 MBq of 68Ga-MAA was intravenously injected, and 4D PET Q imaging was performed in the same field of view. A meta-analysis showed that Q imaging has more potential than V imaging for improving lung dose parameters in RT plans (33).

#### 2.2.2 FLART plans based on PET/CT lung functional imaging

One of the main steps in constructing FLART plans is delineating the FL volume and integrating it into the RT plan as organs at risk (OARs). Currently, the most commonly used method of functional contouring utilizes the relative threshold method based on the maximum pixel value (pmax) and the threshold method relative to the whole lung function (WLF). Based on these two methods, current research has applied V/Q PET/CT to IMRT (17), VMAT (70), and SBRT (71) for FLART studies. Siva and Bucknell et al. (17, 70) used a visual adaptation method with a 70% SUV threshold to divide the lung into high Q lung capacity (HPLung) and high V lung capacity (HVLung). Nevertheless, Siva et al. found HVLung was much smaller than HPLung, so they derived V lung volume (VLung) as an approximation of HPLung volume, as previously reported by Munawar et al. (48). The results showed that the MLD of HPLung decreased by 13.0%, and the V_5_, V_10_, and V_20_ doses decreased by 13.2%, 7.3%, and 3.8%, respectively. The 50% SUV threshold, however, resulted in many small-volume regions throughout the entire lung rather than a continuous volume, limiting RT’s adaptive capabilities. Therefore, the selection of a threshold for FL definition is crucial, and there is currently no clear indicator to define an appropriate threshold. Recently, Pinot et al. (72) developed a relative whole-lung functional threshold segmentation method for PET/CT Q imaging that allowed overlapping contours and was relatively unaffected by hotspots, providing reliable and consistent functional volumes. FL volume was defined as the minimum volume within the anatomical volume that contained 50%, 70%, and 90% of total activity. By prioritizing protection of the FL region, SBRT planning significantly reduced MLD and the percentage of lung volume receiving 5-20 Gy.

#### 2.2.3 Clinical benefits from FLART based on PET/CT lung functional imaging

The evaluation of lung Q is particularly relevant to RILI because the vascular endothelium, like lung cells, is considered to be one of the most sensitive tissues in the lung to radiation (73). Preserving intact lung parenchyma Q during RT may reduce radiation-induced vascular damage and reduce the risk of RP. Previous studies have reported a strong correlation between PET/CT lung function and pulmonary function testing (PFT) parameters (74). Furthermore, PET/CT diagnosed pulmonary embolism (PE) is more accurately than conventional lung V/Q scanning and identifies changes in lung function during RT, allowing timely adjustments to the RT plan (75, 76). A recent study validated the feasibility of using 68Ga-4D-V/Q PET/CT during RT for ART (70). According to the study, FL volume may change during the treatment process, and some patients may benefit from adjusting their RT plans in the fourth week of treatment to preserve the high-Q or high-V lung regions. This study suggests that FL volume changes during the treatment process. Further prospective research is needed to determine which patients can benefit the most from this mid-term adjustment.

### 2.3 Current problems and research direction of nuclear medicine image-guided FLART

Although lung function requires the Q and V of alveoli, V abnormality is more common than Q abnormality in the lungs (17). V images contained more noise than Q due to significantly reduced lung activity (70). Unless more specific indicators were available, Q imaging could reasonably be used to assess lung function. According to Forghani et al. (77), 25% of stage III lung cancer patients had low consistency on SPECT/CT V/Q. Another study found significant differences in response to V and Q doses in 20–30% of patients (78). Kimura et al. (79) found greater clinical correlation with RP using combined Q/V imaging than when using only V or Q imaging. To promote real lung function, it is necessary to maintain an appropriate ratio between V and Q. There are also potential drawbacks to V/Q SPECT technology. The most significant problem is the difficulty in image registration, especially in the lungs, where patients breathe during SPECT and CT imaging, leading to differences in the two images (22). PET/CT combined with 4DCT improves image quality and registration accuracy (61, 63, 64). In addition, a certain area of the lungs may experience reduced functionality due to proximal vascular obstruction. A lung region might regain functionality after the obstruction is removed during RT (22, 80). In this case, DFH underestimated radiation’s functional impact. Therefore, prospective clinical experiments will be needed to examine how FL changes during RT, and when RT plans need to be adjusted. Summary of the study on FLART based on nuclear medicine functional imaging is presented in **Table 1**.

**Table 1:** Detailed description of nuclear medicine-guided RT research, including patient characteristics, planning techniques, and clinical benefits.

Reference	Patients	Characteristics	Imaging type Planning technique	Definition FL	Benefit of FL sparing (% difference between means)
Siva et al. (17)	20	Age (med) 68 NSCLC 100% Stage I-II 35% Stage III 55% Stage IV 10%	PET/CT V Gallium-68 Q ^68^Ga-MAA IMRT	High V/Q 70% of max V/Q 50% of max	** fMLD (Q) ↓ 1.7 Gy, fV_5_ Gy (Q) ↓ 13.2%, fV_10_ Gy (Q) ↓ 7.3%, fV_20_ Gy (Q) ↓ 3.8%.
St-Hilaire et al. (24)	15 (13 patients accepted SPECT Q)	Age NS NSCLC 53% SCLC 20% Stage III 100%	SPECT Q ^99m^Tc-MAA IMRT	SPECT cost function.	** Median reduction: fMLD ↓ 0.9 Gy, fV_10_ Gy ↓ 2.2%.
McGuire et al. (37)	5	Age NS NSCLC NS Stage NS	PET/CT Q ^99m^Tc-MAA IMRT	According to the degree of regional perfusion, the lung is divided into four lung zones.	fV_20_ Gy ↓13.6%, fV_30_ Gy ↓10.5%.
Wang et al. (43)	39	Age (med) 61 NSCLC 100% Stage III 100%	SPECT Q ^99m^Tc-MAA IMRT	30% of max.	** fV_10_ Gy ↓ 5.21%, fV_20_ Gy ↓ 4.25%, fV_30_ Gy ↓ 2.38%, fV_35_ Gy ↓ 10.5%.
Siva et al. (44)	14	Age (med) 66 NSCLC 100% Stage I–II 43% Stage III 50% Stage IV 7%	4D PET/CT Q ^68^Ga- MAA 3DCRT	Perfused: all lung with uptake, well Q 70% of max	** Median reduction: fMLD ↓0.8 Gy, fV_20_ Gy ↓5.3%.
Yin et al. (45)	10	Age NS NSCLC 100% Stage NS	SPECT Q IMRT vs 3DCRT	30% of max	** 3DCRT: fV5 Gy ↓ 6.50%, fV_20_ Gy ↓ 14.02%, fV_30_ Gy ↓ 22.30%; IMRT: fV5 ↓ 3.05%, fV_20_ Gy ↓ 14.16%, fV_30_ Gy ↓ 4.87%.
Shioyama et al. (47)	16	Age (med) 62 NSCLC 100% Stage III 69% Stage IV 19%	SPECT Q ^99m^Tc MAA IMRT	50% and 90% of max.	** FL90: fV5Gy ↓ 11.7%, fV_10_ Gy ↓ 12%, fV_20_ Gy ↓ 6.8%.
Munawar et al. (48)	10	Age NS NSCLC 100% Stage III 100%	SPECT V ^99m^TcTechnegas IMRT	50% and 70% of max	Optimization to SPECT V caused; FMLD by 3 Gy if <5% of FL50 overlapped PTV.
Lukovic et al. (49)	21	Age NS NSCLC 100% Stage III 100%	SPECT V IMRT	70% of max.	fV_10_ Gy (HV) ↓ 4.5%, fV_20_ Gy (HV) ↓ 3%.
Christian et al. (52)	6	Age NS NSCLC 100% Stage NS	SPECT Q ^99m^Tc-MAA 3DCRT	80% of the max.	fV20 Gy ↓ 16% (One patient with bilateral upper lobe perfusion deficits.)
Bucknell et al. (70)	25	Age NS NSCLC 100% Stage I–II 32% Stage III 24% Stage IV 20%	^68^Ga-4D-V/Q PET/CT VMAT	A 70th centile threshold	** fV_5_ Gy ↓ 2.5% (HQ), ↓ 2.1% (HV); fV_20_ Gy ↓ 0.9% (HV)
Lucia et al. (71)	60	Age (med) 69 NSCLC 42% Stage NS	PET/CT Q ^68^Ga-MAA SBRT	50%, 70%, and 90% of the max.	** A significant reduction of the MLD and V_5_ Gy to V_20_ Gy in all functional volumes.
Lee et al. (81)	8	Age (med) 72.5 NSCLC 100% Stage II 12.5% Stage III 87.5%	SPECT/CT Q ^99m^Tc-MAA VMAT, PBS	70% of max.	** VMAT: fMLD ↓ 7.6 Gy, PBS VS VMAT: fMLD ↓ 3.7Gy, fV_5_ Gy ↓ 27%, fV_10_ Gy ↓ 17%

**, denotes statistically significant result; Q, perfusion; V, ventilation; HQ, high perfusion areas; HV, high ventilation areas; NS, non-specified; FL, functional lung; fV_x_, functional volume receiving ≥ x Gy; V_x_, lung volume receiving ≥ x Gy; fMLD, functional mean lung dose; MLD, mean lung dose; ^99m^TC-MAA, technetium Tc-99m albumin aggregated; ^68^Ga-MAA, human albumin macroaggregates labeled with Ga-68; RP, radiation pneumonitis; Med, median.

## 3 CT image-guided FLART

Using inhalation gas tracers or intravenous contrast agents for imaging can result in increased radiation exposure and scan times (65). While CT-based V imaging provides additional benefits by providing functional information without additional imaging tests (16, 80). The most common CT techniques used in lung functional imaging research include 4DCT (14, 16, 19, 26, 44), multi-detector CT (MDCT) (82, 83), dual-energy CT (DECT) (84, 85), and CT-enhanced imaging, which uses inert gases. In the past, CT imaging was slow and difficult to apply in clinical practice. With advances in imaging technology, MDCT, which includes multiple detector arrays, has improved imaging quality, scanning modes, and clinical applications (86, 87). However, compared to 4DCT and DECT, MDCT has certain limitations in terms of imaging quality, tissue resolution, radiation dose, respiratory motion artifacts, and tissue composition analysis (88–90). Therefore, 4DCT and DECT are gradually replacing MDCT as preferred choices in clinical practice. Use of CT lung functional imaging for the FLART project research as shown in **Table 2**.

**Table 2:** Detailed description of CT-guided RT research, including patient characteristics, planning techniques, and clinical benefits.

Reference	Patients	Characteristics	Imaging type Planning technique	Definition FL	Benefit of FL sparing (% difference between means)
Huang et al. (11)	36	Age (med) 66 NSCLC 100% Stage II 8% Stage III 87% Stage IV 5%	XeCT V IMRT 47% VMAT 53%	Visually defined.	** Mean relative reduction 29% for ≥G2 RP. fV_20_ Gy ↓ 12%, fMLD ↓ 13%
Faught et al. (13)	70	Age NS NSCLC 100% Stage I-II 24% Stage III 70%	4DCT V IMRT 59% 3DCRT 41%	15% of max.	** Mean absolute reduction 7.1% for ≥G2 and 4.7% ≥G3 RP.
O’Reilly et al. (15)	74	Age NS NSCLC(100%) G2+ RP 33% Stage III 100%	4DCT V Photon 50% Proton 50%	6%, 45%, and 60% of max.	** fV_20_Gy (HV) was a significant indicator for RP.
Yaremko et al. (18)	21	Age (mean) 69 NSCLC 100% Stage III 100%	4DCT V IMRT	90% of max.	** fMLD ↓ 2.9 Gy, fV_5_ Gy ↓ 16.6%, fV_10_ Gy ↓ 11.7%, fV_20_ Gy ↓3.4%
Waxweiler et al. (19)	25	Age NS NSCLC 100% Stage III 100%	4DCT V VMAT	Auto segmentation of any lung with no less than a 15% reduction in V	** Structure based: fMLD ↓ 2.8 Gy, fV_5_ Gy ↓ 13.7%, fV_10_ Gy ↓ 14.9%, fV_20_ Gy ↓ 5.6%, fV_30_ Gy ↓ 2.9%
Yamamoto et al. (25)	1	Age NS NSCLC 100% stage III 100%	4DCT V VMAT	FL regions based on image/voxel method.	** fV_20_ Gy ↓ 5%
Yamamoto et al. (26)	15	Age (mean) 75 NSCLC 100% Stage I-II 73% Stage III 45% Stage IV 7%	4DCT V IMRT, VMAT	Three FL regions based on probability density function.	** IMRT: fV_20_ Gy ↓ 3.5% fMLD ↓ 1.8 Gy; VMAT: fV_20_ Gy ↓ 2.3% fMLD ↓ 2 Gy.
Li et al. (53)	17	Age (med) 67 NSCLC 100% Stage III 29.4% Stage IV 70.6%	4DCT V HT	50%, 40%, 30%, 20%, and 10% of Max.	** Mean absolute reduction: Risk of Func_planTop40/50 ↓ 7.39%/8.6%. FV_5_ Gy, fV_20_ Gy, and fMLD of Func_planTop30/40/50, and fV_10_ of Func_planTop40/50 were significantly lower.
Vinogradskiy et al. (80)	118	Age NS NSCLC 100% Stage I 36% Stage III 64%	4DCT V SBRT	FL regions based on probability density function.	fMLD (Stage III) ↓ 0.8Gy, fMLD (Stage I) ↑ 1.1Gy.
Bahig et al. (85)	20	Age NS NSCLC 68% Stage I 72% Stage III 28%	DECT Q SBRT, IMRT	Lung volume was divided into six different functional sub-regions based on iodine concentration.	** Absolute reduction: IMRT: fMLD ↓ 1.5Gy, V_20_ Gy ↓ 3%; SABR: fMLD ↓ 0.5Gy, V20 Gy ↓ 1%.
Yamamoto et al. (91)	14	Age (med) 74 NSCLC 100% Stage III 100%	4DCT V IMRT (adaptive or non-adaptive)	FL regions based on image/voxel method.	** fMLD (adaptive) ↓ 5%, fMLD (non-adaptive) ↓ 3.6%.
Vinogradskiy et al. (92)	67	Age (mean) 65 NSCLC 79% SCLC 21% Stage I-II 12% Stage III 76% Stage IV 12%	4DCT V IMRT	15% of max.	** fMLD ↓ 1.4Gy, fV_5_ Gy ↓ 3.4%, fV_10_ Gy ↓ 6.4%, fV_20_ Gy ↓3.5%, fV_30_ Gy ↓ 1.8%; The rate of grade ≥2 RP was 14.9% (10 of 67 patients).
Utsumi et al. (93)	12	Age (mean) 69 NSCLC 83% SCLC 17% Stage I-II 16% Stage III 83%	XeCT V VMAT	Three equal areas were separated by the FL CT value histogram.	** fMLD ↓ 0.27 Gy (Total function), ↓ 0.32 Gy (High), ↓ 0.27 Gy (Moderate), ↓ 0.2 Gy (Low)
Ieko et al. (94)	13	Age(mean)75.1 NSCLC 92% Stage I-II 77%	4DCT V 3DCRT, VMAT, PT	20% of max.	** Compared with 3D-CRT and VMAT, PT resulted in a significant reduction in fMLD, fV_5_ Gy, and fV_10_ Gy.
Huang et al. (95)	8	Age (med) 69 NSCLC 100% Stage III 75% Stage IV 25%	4DCT V IMRT, IMPT, DSPT	Based on the Jacobian method, the regional V map was obtained to divide the lung volume into three equally volumed functional regions.	** Compared with IMRT, V_5_ Gy(Total lung) ↓ 34% (DSPT), ↓ 38.8% (IMPT); fV_5_ Gy (HV) ↓ 4.3% (IMPT), fV_20_ Gy (HV) ↓ 3.1% (IMPT)
Huang et al. (96)	11	Age (med) 56 NSCLC 55% Stage III 100%	4DCT V IMRT	80%, 70%, and 60% of max	** fV_20_ Gy ↓ 2.7%

**, denotes statistically significant result; Q, perfusion; V, ventilation; HV, high ventilation areas; NS, non-specified; FL, functional lung; fV_x_, functional volume receiving ≥ x Gy; V_x_, lung volume receiving ≥ x Gy; fMLD, functional mean lung dose; MLD, mean lung dose; RP, radiation pneumonitis; Med, median.

### 3.1 4DCT

4DCT imaging was developed to provide tumor motion information and improve RT planning. Further, 4DCT images include variations in air content caused by respiration in the lung parenchyma, which can be used to produce lung V images (18). Images from SPECT V/Q (35–37, 97), PET/CT (17, 29, 44, 98), MRI (99, 100), and PFT (91, 101) confirmed the validity of 4DCT V. Numerous studies have detailed the methods (102, 103) and validation (103, 104) of 4DCT V imaging as well as its potential clinical applications as a functional imaging modality (26, 105–107). Through 4DCT scanning, maximum inhalation and exhalation images can be obtained to generate lung function maps (91) and measure breathing retention (98).

#### 3.1.1 Lung functional imaging based on 4DCT

In the free-breathing state, 4DCT was used to collect ten-time phases of a complete breathing cycle. CT images with phase or amplitude resolution were used to calculate the V for each voxel in the lungs (102, 103, 108). By calculating the local air content change for each voxel, a 3D graph of the V function is generated (19). According to the literature, there are three main 4DCT V-imaging algorithms (VIA), which are based on density (Hounsfield units), Jacobian matrix, and region. Among them, the density VIA was described by Guerrero et al. (109), based on the physical density changes between the exhalation and inhalation peak CT images, using deformable image registration (DIR) and underlying CT density information to generate static 3D V images. Reinhardt et al. (102) introduced the Jacobian matrix VIA, which quantified regional volume changes of lung voxels by utilizing the determinant of the Jacobian matrix obtained from DIR spatial transformation. Besides, Kipritidis et al. (110) proposed a region-based VIA, estimating V situation by evaluating the 4D regional averaged time-product of air and tissue densities at each voxel without DIR. However, the correlation between the V images generated by these algorithms and clinical gold standards [including ^99m^Tc-SPECT (111) and ^68^Ga-Galligas PET (112)] varies significantly (97, 113). According to studies, region-based algorithms have a higher correlation with ^68^Ga-Galligas PET and less variability, but they are prone to motion blur and limited in spatial resolution, possibly limited to lung voxels within HU values of (-1000, -600) (110). Further, region-based algorithms require much more computation time because they require 10 phase matrices, unlike the two other algorithms (114). Compared with the other two algorithms, the Jacobian matrix algorithm is much less accurate (91, 110). While Eslick et al. (115) demonstrated good consistency between CT density changes and PET lung V images. As 4DCT imaging becomes more common in patients with lung cancer, using 4DCT V imaging does not require additional doses or economic costs (92). To confirm its clinical applicability, these clinical trials are investigating the use of 4DCT V imaging to prioritize the protection of high-functioning lung regions (NCT02528942, NCT02773238, NCT02002052, NCT02308709, and NCT02843568).

#### 3.1.2 FLART plans based on 4DCT Lung functional imaging

**Figure 1:**
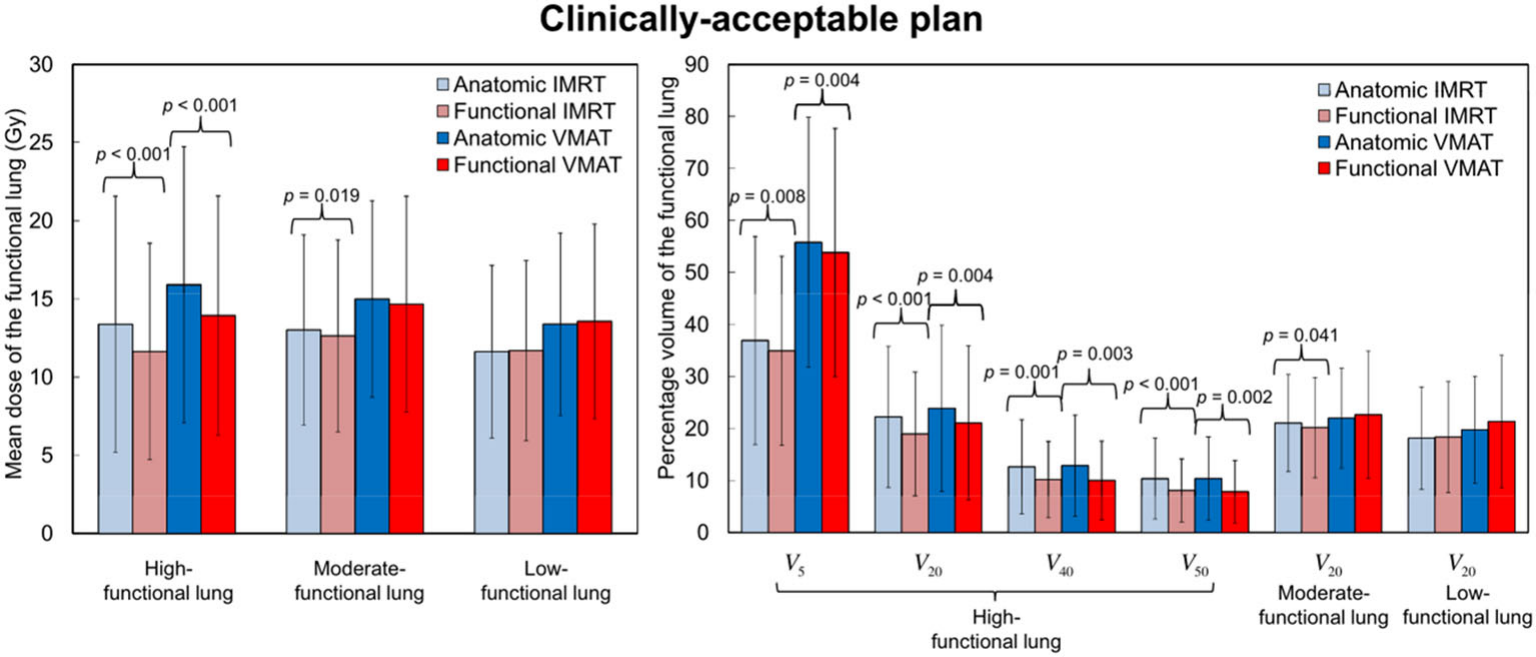
Comparisons between anatomical and functional treatment plans of mean dose (mean ± standard deviation) and percentage of volume receiving ≥5, ≥20, ≥40 or ≥50 Gy (Vx) for the three functional lung regions. Reproduced from reference (26) with permission from Elsevier, copyright 2011.

Treatment plans focused on FL commonly employ optimization techniques based on structure and images. Structure-oriented approaches optimize treatments by setting functional contours based on 4DCT images of lung volume. This methodology has been verified alongside alternative lung functional imaging methods (15–18, 25–27, 100). As functional images are transformed into binary masks, the process of generating functional contours may eliminate heterogeneous functional data (92). By contrast, image-based techniques enable the inclusion of all functional image data in optimization processes through 4DCT images of lung volumes (24, 51). Research has indicated that both structure-based and image-based optimization methods have comparable predictive impacts on RP, with superior performance over standard approaches (116). However, structure-based techniques are compatible with any modern treatment planning system (TPS), but image-based methods need a more sophisticated TPS (19).In structure-based methods, there are two main approaches: the first uses threshold-based segmentation FL. Another method divides the lung into six regions, calculates average V values, and compares them to the overall average to identify functional defect regions (116). Functional defect regions are areas where V decreases compared to a completely homogeneous lung in nuclear medicine (117). Function-based methods commonly use thresholding to partition functional regions, requiring careful consideration of thresholds. Through DIR and quantitative image analysis, V values were computed from 4DCT V images to derive DFH. A functional volume receiving dose is indicated by the vertical axis, while the “hottest” functional volume is indicated by the horizontal axis (118). V values derived from 4DCT V images were transformed into percentiles, enabling the selection of a suitable threshold for categorizing lungs into high or low functional areas (13, 15, 25, 91). Notably, the precise threshold for lung functionality in CTV images remains undefined. In a systematic review and meta-analysis of FLART (119), the range of lung function definitions varied from 20% to 90%. Among these studies, multiple definitions of lung function were used. The most commonly used maximum level lung function percentages were 70% and 30%, followed by 50% and 60%. Literature often categorizes lung tissue into high- or low-functional areas (HFA or LFA) (13, 15, 25, 92, 120). Based on two thresholds, Yamamoto et al. (26) divided the lung into three equally-sized functional areas: HFA (>66%∼100%), intermediate functional lung area (>33%∼66%), and LFA (0∼33%). Result indicated a significant reduction in HFA dosage, but no improvement in intermediate functional areas or LFA dosage, with a majority of the dosage transferred from HFA to intermediate functional areas and LFA (**Figure 1**). By utilizing the FLART plan to evade HFA, the treatment dose is minimized to the greatest extent possible; however, no specific strategy was employed to safeguard LFA (121). Yamamoto et al. (25) noted that the decrease in HFA parameters was not a result of an overall reduction in lung dose, but rather a deliberate avoidance of HFA. Planning strategies focused on avoiding HFA may lead to an escalation in the dose to the LFA, which still retains some level of functionality. Once the dose surpasses a certain threshold, the resulting damage becomes irreversible.

#### 3.1.3 Clinical benefits from FLART based on 4DCT Lung functional imaging

By acquiring functional data directly from a patient’s 4DCT simulation, 4DCT V provided a distinct advantage over SPECT imaging in treatment planning (16, 80). Moreover, the higher resolution of 4DCT V images simplifies the process of image fusion compared to SPECT (122), thus increasing its acceptance by clinicians and lung cancer patients. With the use of 4DCT-based functional planning, radiation doses are reduced in various treatment plans, such as IMRT (16, 25–27, 35, 48), VMAT (16, 26, 81, 93), and PT (94, 95). In plans using functional planning of IMRT and VMAT, fMLD was significantly reduced by 1.8 Gy and 2.0 Gy, respectively (26). Several FLART studies have shown that the mean reduction in f-MLD can be 2.5 Gy, with a maximum reduction of up to 10 Gy (17, 18, 24, 26, 48). Additionally, fV_5_ can be decreased by 10-16% (17, 18, 96) and fV_20_ by 3-9% (37, 47, 99, 123). Ieko et al. (94) conducted a study evaluating the impact of 4DCT V imaging-guided PRT on SBRT, comparing it with VMAT and 3D-CRT. PRT resulted in significant reductions in f-MLD and fV_5_. Clinical findings indicate that fV_20_ and f-MLD are the most predictive indicators of RP (**Table 3**) (19, 119). FLART has been shown to reduce the incidence of ≥2-grade RP from 25% to 12% compared to historical controls (92).

**Table 3:** Standard and functional measurements of individuals with RP (grade 0-1) and RP (grades 2–5).

Parameter	Standard			Functional segmented			Functional SPECT-weighted		
RP group	Grade 0–1	Grade 2–5	p	Grade 0–1	Grade 2–5	p	Grade 0–1	Grade 2–5	p
MLD (Gy)	11.1	14.33	**0.01**	8.04	16.56	**0.01**	9.92	15.95	**<0.01**
V5 (%)	41.05	46.9	0.14	38.66	48.36	**0.01**	40.77	50.66	**0.03**
V10 (%)	28.27	34.02	0.07	22.74	36.24	**<0.01**	26.17	37.1	**0.01**
V20 (%)	18.98	25.48	**0.01**	11.61	27.83	**<0.01**	16.23	26.76	**<0.01**
V30 (%)	13.34	19.4	**<0.01**	8.13	23.86	**<0.01**	10.92	21.66	**<0.01**
Parameter	Standard			Functional segmented			Functional SPECT-weighted		
	OR	95% CI	p	OR	95% CI	p	OR	95% CI	p
MLD (Gy)	0.82	0.63–1.063	0.13	1.53	1.2–2.0	**<0.01**	1.4	1.1–1.8	**<0.01**
V5 (%)	0.97	0.9–1.05	0.5	1.1	1.01–1.2	**0.02**	1.1	0.99–1.1	0.05
V10 (%)	0.95	0.86–1.06	0.41	1.1	1.03–1.2	**0.01**	1.1	0.99–1.1	0.05
V20 (%)	0.94	0.84–1.05	0.25	1.2	1.04–1.3	**<0.01**	1.1	1.02–1.2	**0.02**
V30 (%)	1.12	1.0–1.3	**0.04**	1.2	1.1–1.4	**<0.01**	1.2	1.1–1.4	**0.01**

MLD, mean lung dose; V*x*, volume of lung receiving; OR, odds ratio; CI, confidence interval; RP, radiation pneumonitis. Reproduced from reference (55) with permission from Elsevier, copyright 2015. Significant p-values (p < 0.05) are indicated in bold.

### 3.2 DECT

Recent research has not only utilized 4DCT for FLART planning but also evaluated the feasibility of DECT in FLART planning (84, 85). The DECT setup comprises two X-ray tubes generating high- and low-energy X-ray beams, respectively. By employing these X-ray beams with varying energies, two sets of CT images are acquired from scanning the target. Materials with different decay coefficients can be identified and quantified by X-rays of different energies. Prior studies have primarily used DECT for diagnostic purposes, differential diagnosis, characterization of tumor differentiation and gene expression, staging, and the assessment of prognosis (124). Serving as an innovative imaging technique, DECT surmounts traditional CT constraints in tissue characterization (125, 126). Furthermore, the administration of iodine contrast agents enhances DECT imaging for lung Q assessment, while inert gases like xenon (Xe) and krypton (Kr) are utilized for lung V imaging (84, 127–129).

#### 3.2.1 Lung functional imaging based on DECT

Quantification of lung Q involves the measurement of pulmonary blood volume (PBV), which is delineated as the proportion of capillary volume relative to lung mass (130). ^68^Ga-MAA PET/CT imaging accurately assesses PBV. Nonetheless, PET/CT imaging methodologies are more advanced and unavailable in many medical facilities, restricting their widespread application. Gaudreault et al. (131) demonstrated that DECT imaging enhanced by iodine contrast agents could serve as a substitute for PET/CT imaging in evaluating lung Q. In the study by Kerl et al. (132), the operation procedure for DECT Q imaging was described. During injection, a high concentration of iodine-based contrast agent is injected within a specified time by optimizing injection parameters. A mixture of contrast agent and physiological saline is injected in stages at a specific rate. Finally, DECT images are acquired during the late arterial phase. Additionally, Bahig et al. (85) generated iodine concentration maps using the double material decomposition technique. The method leverages the dual energy information from DECT images to compute iodine concentration through the establishment of a calibration curve for the production of the lung Q agent map. Research has shown that DECT is a reliable alternative to the direct measurement of local Q in lung parenchyma (133, 134). Even so, iodine contrast agents can pose challenges related to precision and consistency, such as artifacts and patient-specific injection dosages (135–137).

Apart from enabling iodine contrast-enhanced Q imaging with DECT, it can also facilitate V imaging with an enhanced Xe and Kr. Xe, a stable and radiopaque inert gas naturally found in the atmosphere. With inhaled Xe-enhanced DECT (Xe-DECT), three-material decomposition is utilized to differentiate Xe from other substances (such as air and soft tissue) and to describe and quantify V abnormalities in terms of absorption characteristics (138–140). Xe-DECT can be performed using dynamic or static scanning protocols (140). Dynamic CT scans are generated by providing high levels of oxygen (O_2_) followed by a mixture of 30% Xe and 70% O_2_ to maintain consistent coverage throughout the wash-in and wash-out phases of Xe (141–143). Conversely, the static acquisition protocol involves capturing images of the entire lung at distinct time intervals. Notably, the static DECT method is prized for its significant reduction in radiation exposure (141, 144). Nonetheless, many researchers preferred the dynamic scanning method for assessing V dynamics by tracking changes in contrast gas concentrations and distributions (138, 143). In addition to displaying normal lung V maps, Xe-DECT can evaluate regional lung V in chronic obstructive pulmonary disease (COPD), asthma, bronchiolitis obliterans, bronchial atresia, and PE (138). However, Xe-DECT was limited in clinical settings by radiation exposure and adverse effects, including drowsiness, headache, and nausea (145).

Research has explored stable Kr or a combination of Xe and Kr for lung V imaging to mitigate the high costs and side effects of Xe (128, 146). Kr concentrations exceeding 70% were shown to be effective in replacing Xe in DECT lung V imaging in rabbits, although drawbacks were also noted (147). Previous studies on conventional CT and DECT have shown that Kr can serve as an inhalation contrast agent for human and animal radiographic imaging (128, 147, 148). Similar to Xe-DECT, patients inhale a mixture of Kr and O_2_ in a certain ratio and hold their breath during DECT scanning while taking a deep breath. By differentiating substances, Kr V images with varying degrees of enhancement can be obtained. This indicates changes in the V situation through CT density changes. Using 80% Kr and 20% O2 as a contrast agent for DECT V imaging was found to be well tolerated by patients with severe emphysema by Hachulla et al. (128). The results indicated significantly higher attenuation levels in the normal lung than in the emphysematous region, suggesting Kr is a promising contrast agent. As a contrast agent, Kr has some inherent disadvantages, such as certain radioactive properties, a relatively short half-life, and a lower atomic mass, which makes Kr less stable than Xe. Therefore, the assessment of the local V situation poses certain difficulties (149).

#### 3.2.2 FLART plans based on DECT Lung functional imaging

The use of Xe and Kr for lung V imaging has been demonstrated in prior studies, but mostly in research settings. FLART planning with Xe and Kr for DECT V imaging has limited studies due to limitations and side effects. Studies have shown that DECT-iodine mapping can provide extensive functional data in patients undergoing lung RT, aiding FLART planning through systematic and personalized optimization (85). Studies have confirmed the association between iodine-enhanced DECT V imaging and PET (127, 131). Functional segmentation is a critical component of FLART planning. Bahig et al. (85) conducted a study in which pulmonary iodine images were obtained via DECT scanning. The lung function was divided into six equally spaced subregions according to the range of iodine concentration from minimum to maximum. Each subregion was treated as a weighted structure (OARs) influencing overall lung function via weight modifications. The results showed a strong correlation between the relative lung lobe function measured by DECT and the results obtained from SPECT/CT imaging. The average differences between V_5_ and MLD in anatomical and FL volumes were 16% (p = 0.03) and 15% (p = 0.047), respectively. Lapointe et al. (84) conducted a study where the lung was manually divided into five subregions comprising the upper and lower lobes of both the left and right lungs. The differential function (δF) of each subregion compared to the entire lung was determined based on iodine concentrations assigned to individual voxels within the functional area. They found strong agreement between SPECT/CT and DECT images in terms of calculated differential functions for lung subvolumes. This underscores the promising potential of employing DECT in RT to preserve FL tissue. There was currently limited research on using DECT for FLART. Future research is hoped to verify its feasibility for the FLART program and explore its potential clinical benefits.

#### 3.2.3 Clinical benefits from FLART based on DECT Lung functional imaging

By scanning the treatment site with contrast agent, the DECT-iodine map can be obtained directly for RT plan, providing precise anatomical and functional correlation without additional radiation exams. Research demonstrated that DECT-iodine mapping effectively identified Q defects in PE (150–152) and lung parenchymal diseases (153, 154). Pansini et al. (153) examined DECT-iodine maps in 57 patients (including 37 with emphysema). They found that areas with decreased iodine distribution correlated significantly with emphysema. The severity of Q changes was related to parenchymal damage. In addition, Aoki et al. (155) established the effectiveness of DECT arterial stage iodine uptake as a predictive biomarker for lung cancer recurrence after SBRT. In the case of radiation damage changing over time, DECT Q imaging provides similar information to PET Q imaging and is an economical alternative to PET imaging (127). Despite the limited clinical validation of DECT in RT, the literature acknowledges its potential applications, including improved dose calculation accuracy (156, 157), decreased metal artifacts (158), and enhanced tumor delineation and tissue characterization (159, 160).

### 3.3 Current problems and research direction of CT image-guided FLART

Research indicates that about 70% of lung cancer patients exhibit regional functional variability that favors functional avoidance (35, 52). There are two important factors for FLART: functional structural heterogeneity and spatial dose distribution. When a patient’s lung function is consistent, no particular area requires prioritized protection. Functional avoidance may preferentially expose the defective area instead of the functional region, if a patient shows a heterogeneous image with significant Q and V defects (35). An assessment of functional defect size is necessary for evaluating the relevance of functional information in treatment planning, particularly when the defect exceeds approximately 25% of total lung volume. Study by Katsuta et al. (16) indicated that DFH calculation was frequently performed in advanced NSCLC, especially for patients with significant irradiation lung volumes and a greater rate of RP. And in advanced-stage NSCLC RT, patients with FL in close proximity to the tumor are particularly suitable for RP prediction using DFH characteristics (161). Yamamoto et al. (26) showed that FLART performed best in patients with substantial overlap between FL and PTV, whereas it performed poorly in patients with less overlap. However, 4DCT V imaging does have limitations, such as inaccurate registration (162) and numerical instability (163), as well as suboptimal correlation with other lung functional imaging techniques (97). 4DCT functional imaging is also limited by its focus on V volume calculation without providing Q information. However, research indicated that approximately 20% of lung cancer patients can exhibit varying V and Q distributions (77).

Currently, DECT can simultaneously acquire both lung V and Q data. Nevertheless, using Xe and iodine contrast agents to acquire V and Q DECT images simultaneously might increase radiation exposure and prolong scanning time (164, 165). Similarly, using both Xe and iodine enhancement in a single scan presents difficulties because their atomic numbers are close (iodine at 53, Xe at 54), suggesting similar DECT spectral attenuation properties (166). Moreover, research exists on Kr and iodine-enhanced DECT, since iodine exhibits significant attenuation variations with increasing contrast agent doses in CT imaging, suggesting a potentially significant impact on Kr analysis based on substance decomposition theory (129). Further research is required to determine and optimize the dose of contrast agents in mixed contrast. Iodinated contrast agents are mandatory for Q DECT implementation, which limits its applicability in patients with contraindications. Furthermore, the presence of an iodine threshold for functional zoning requires additional clinical evidence. A functional imaging approach reveals significant differences in DFH compared with traditional anatomical dosimetry, which could clarify the limitations of conventional DVH parameters. The future research aims to improve lung function preservation with weighted functional volumes.

## 4 MR image-guided FLART

MRI offers several benefits over CT, including detailed soft tissue images, lowering radiation exposure, and enabling multi-angle analysis of tissue (65, 120). Nevertheless, lung MRI presents many challenges due to its low proton density, rapid signal decay (caused by multiple air-soft tissue interfaces), and motion artifacts (167). The advancement of imaging technology has led to the development of new MRI techniques aimed at addressing these limitations. Several recent studies have demonstrated the feasibility of lung MRI assessment with Fourier-resolved MRI (FD-MRI) (168) (169) and MRI using hyperpolarized (HP) gases (169, 170), fluorinated gases, and oxygen (171, 172).

### 4.1 Lung functional imaging based on MR

FD-MRI represents a novel method for the simultaneous non-contrast-enhanced imaging of lung V and Q (169). This technique utilizes rapid 2D TrueFISP pulse sequences to efficiently capture lung images, removing the need for ECG or respiratory triggers. A non-rigid image registration algorithm is employed to address respiratory motion. Furthermore, the Fourier analysis of time variations in image intensity can distinguish between blood signals and lung parenchyma signals, enabling the computation of V and Q-weighted images. Compared to traditional methods that utilize contrast agents or radiotracers, this method provides considerable advantages. There is a significant correlation between FD-MR imaging and short-term reproducibility of PFTs (173). An animal experimental study assessing regional lung V and blood flow Q confirmed the consistency in qualitative assessment between FD-MR imaging and SPECT/CT (174). However, FD-MRI provides only indirect information about V and Q, and its sensitivity may be inferior to that of directly imaged HP gas MRI (175).

HP gas MRI accurately determines the location and severity of V deficiencies in the lungs (176). The commonly used HP gases for MRI research are ^3^He and ^129^Xe gases. These gases serve as inhalable MRI contrast agents and can provide rich supplementary information about lung function and lung microstructure (177, 178). HP ^3^He MRI serves as a valid alternative to SPECT for the evaluation of V (16, 27). Smith (20) and Hughes et al. (179) have provided comprehensive protocols for the acquisition of MRI data utilizing ^3^He. The high-pressure treatment of ^3^He gas using spin-exchange optical pumping technology can substantially increase the magnetization of ^3^He by a factor of 104 to 105. The patient was directed to inhale 1 liter of a mixture comprising ^3^He and medical nitrogen (N_2_). Static volume images of ^3^He were acquired using rapid gradient echo imaging over a period of 16 seconds. Matthew et al.’s study (180) provides qualitative and quantitative evidence for 3He MRI and 4DCT V maps, showing good spatial consistency. HP gas ^3^He MRI may not be widely used due to cost and limited global helium supplies.

In comparison to ^3^He, ^129^Xe has significant natural reserves. This guarantees stable long-term availability and strengthens its economic viability. In addition, ^129^Xe has a high solubility and can dissolve in lung tissue and blood, forming a “dissolved phase.” During the dissolution process, approximately 200 ppm of chemical shifts are produced, and MRI technology can separate the gaseous ^129^Xe from the dissolved ^129^Xe for imaging (181). The latest MRI technology can obtain 3D ^129^Xe images of lung parenchyma, blood, and alveoli in a single breath-hold scan (182). This allows for simultaneous lung V, Q, and gas exchange assessment. To perform ^129^Xe MRI, HP ^129^Xe gas needs to be prepared first. The patient needs to inhale a mixture of ^129^Xe and N_2_ gases and undergo a 3D radial pulse sequence acquisition within a breath-hold time of 15 seconds to obtain distribution images of gaseous and dissolved phase ^129^Xe (171). By using HP ^129^Xe MRI technology, non-invasive information about lung V and gas exchange can be obtained, providing a valuable physiological basis for functional planning in RT.

Recent advancements indicate that, alongside HP gas, fluorinated gas and O_2_ are also applicable for functional imaging in MRI. Fluorinated gas MRI (^19^F-MRI) generally requires patients to inhale a mixture of standard O_2_ with perfluoropropane (PFP) (183) or sulfur hexafluoride (SF_6_) (184), followed by imaging conducted during breath-holding (171). In contrast to HP ^3^He/^129^Xe gas MRI, ^19^F-MRI does not necessitate prior treatment of the gas with hyperpolarization and utilizes non-toxic, naturally abundant fluorinated gases as contrast agents. It is capable of conducting multiple breath imaging and analyzing the inhalation/exhalation kinetics of the gas (185–187). Currently, ^19^F-MRI is restricted to V imaging. Enhancements in image quality, signal-to-noise ratio, and additional repeatability and validation studies are necessary to solidify its evidence base for clinical applications. Oxygen-enhanced MRI (OE-MRI) is a technique that utilizes the influence of O_2_ on the T_1_-weighted signal intensity of the lungs to assess lung function (188, 189). T_1_-weighted images are acquired under standard breathing conditions with 21% O_2_, followed by the patient inhaling 100% pure oxygen for subsequent T_1_-weighted imaging. The distinction between the two indicates the conditions of lung V and oxygen supply (172, 190).

### 4.2 FLART plans based on MR lung functional imaging

In MRI studies of functional imaging, HP gas MRI is the sole method employed for the FLART plan. FLART uses 3He MRI scans to acquire 3He static V images, providing respiratory function information and delineating functional lung regions. Semiautomatic k-means clustering is used to identify and segment V defects, while functional region information is incorporated into the RT plan to prevent irradiation of FL as well (180, 191). Yaremko et al. (192) defined the moderately-to-highly V region as normal V lung (NVL), while V lung (VL) encompassed the entire lung. The FL volume was delineated from breath-hold CT deformation to the planning CT. The findings indicated that in FLART plans, V_20_ and MLD of VL and NVL were significantly decreased by 3.0 ± 0.8%, 3.5 ± 1.0%, 1.0 ± 0.5 Gy, and 1.2 ± 0.7 Gy, respectively. In the existing literature, FLART plans using ^3^He MRI are unproven for clinical outcomes and prognosis, necessitating further study. The aforementioned method is capable of generating both the gaseous and dissolved phases of 129Xe. According to the signal intensity of ^129^Xe MRI images, Ding et al. (171) classified lung volume into “V regions” and “V defect regions.” The RT plan prioritized the reduction of radiation dose to the V regions. The finding indicated that emphasizing the reduction of radiation dose in the V regions derived from ^129^Xe MRI gas exchange distribution maps could markedly decrease indices such as V_5_, V_10_, and V_20_, consequently lowering the risk of RILI. Rankine et al. (191) normalized the V and gas exchange images using linear scaling and calculated percentile values for voxels within and outside the lung. A voxel value within the lung was considered 99th percentile, while a voxel value outside of the lung, such as background, was designated as 1%. A threshold with a relative function interval of 10% was used to generate equal functional contours, which were then transferred to the planning CT via deformable registration. Compared to V-based FLART plans, gas exchange-based FLART plans decreased the dose more effectively to regions with high gas exchange and may significantly lower the incidence of RILI. Gas exchange metrics improve plan quality evaluation moderately over V-guided planning. A preliminary study revealed a moderate correlation (R = 0.53 ± 0.02) between lung ^129^Xe uptake in the blood prior to RT and ^129^Xe V in three patients with NSCLC, which diminished to R = 0.39 ± 0.07 following treatment (170). Additionally, notable differences were observed in the effective uniform dose, V_20_, V_10_, and V_5_, with values of 1.5 ± 1.4 Gy, 4.1 ± 3.8%, 5.0 ± 3.8%, and 5.3 ± 3.9%, respectively. Thus, V alone may not represent actual regional lung function and FLART may have limitations when using V images independently. A precise lung function assessment using 129Xe-MRI is recommended for optimizing RT.

### 4.3 Clinical benefits from FLART based on MR lung functional imaging

FD-MRI eliminates contrast agents and breath-holding, resulting in reduced operational costs and enhanced patient comfort. It possesses significant potential for the diagnosis and monitoring of lung diseases (193, 194). Research indicated that this method may be utilized in the diagnosis and treatment assessment of lung diseases, including lung cancer and PE, offering non-invasive lung functional data for clinical application (175). With inhaled MRI contrast agents, such as HP gases ^3^He and ^129^Xe, lung structure and function can be better understood, enabling disease classification, treatment response assessment, early diagnosis, and long-term monitoring (195, 196). Furthermore, HP gas V MRI can detect airway obstruction and show significant V changes in emphysema and more advanced COPD (197, 198). Research has demonstrated a correlation between ^129^Xe gas exchange function and abnormal carbon monoxide diffusing capacity (DLCO), a clinically recognized gas exchange indicator (199, 200). As a result of its low operating cost and lack of costly polarization devices, ^19^F-MRI is increasingly used in clinical research. ^19^F-MRI allows repeated imaging of breath and gas inhalation and exhalation dynamics analysis. A significant correlation has been found between 19F-MRI-measured V defects and FEV1 in COPD cohorts (201). Conversely, OE-MRI is applicable in the detection of multiple lung diseases, including asthma (202), COPD (203), and interstitial lung diseases (204), as well as in assessing the effects of bronchodilators and inhaled corticosteroids (205). It demonstrates comparability with quantitative CT in evaluating lung functional damage and disease severity (202–204). According to the existing literature, HP gas MRI is applicable in FLART planning and demonstrates potential for enhancing the dosage parameters of FL.

### 4.4 Current problems and research direction of MR image-guided FLART

The research summary on the MRI-guided FLART project is presented in **Table 4**. Research demonstrates a weak to moderate correlation between V and local red blood cell displacement. Defining lung function as V or red blood cell displacement reveals notable differences in dose falloff histogram and effective uniform dose (170). Gas exchange information is distinct from V information, and this significant finding will inform future research in this area. This approach will facilitate future prospective trials that directly compare these two functional planning techniques and assist in identifying which patients are most likely to benefit from gas exchange-guided FLART. Furthermore, research indicated that a reduction in red blood cell displacement, defined as the ratio of regional gas exchange to V, correlates with clinical deterioration (206). In light of the fact that Xe must reach the alveoli before interacting with red blood cells, integrating alveolar gas exchange data into functional indices would improve their predictive capability for RILI, potentially matching or surpassing V-weighted indices. In spite of this, a gas exchange-weighted index that predicts symptomatic RP better than DVH remains undeveloped, suggesting future research opportunities. There have been no studies that have directly compared multiple MRI-based lung functional imaging techniques to identify the optimal method for assessing lung V/Q or evaluating both parameters. Additionally, there should be patient population studies to help clinical practitioners make adaptive selections.

**Table 4:** Detailed description of MRI-guided RT studies for lung function, including patient population characteristics, planning techniques, and clinical benefits.

Reference	Patients	Characteristics	Imaging type Planning technique	Definition FL	Benefit of FL sparing (% difference between means)
Ireland et al. (100)	6	Age NS NSCLC 100% Stage NS	^3^HeMRI V IMRT	FL was defined as the intersection of the lung CT volume with well-ventilated lung segmented from the registered ^3^He images.	** Median reduction: fV_20_ Gy (HV) ↓ 3.1%, fV_20_ Gy (Total) ↓ 1.6%
Rankine et al. (170)	17 (13 healthy volunteers, 1 emphysem)	Age (mean) 68 NSCLC 18% Stage III 18%	^129^XeMRI V, Gas exchange	ROIs were generated to identify the lowest-33%, middle-33%, and highest-33% of each functional volume for V and RBC transfer.	V and gas exchange: the average magnitude of the differences in fEUD, fV_20_Gy, fV_10_Gy, and fV_5_Gy were 1.5 ± 1.4 Gy, 4.1% ± 3.8%, 5.0% ± 3.8%, and 5.3% ± 3.9%.
Ding et al. (171)	10	Age (med) 58 NSCLC 100% Stage NS	^129^Xe MRI V IMRT	Four classes were segmented based on the signal intensity of the HP xenon‐129 images	** fV_5_ Gy ↓ 3.5%, fV_10_ Gy ↓ 2.7%, fV_20_ Gy ↓ 1.5%
Hart et al. (177)	10	Age NS NSCLC 100% Stage NS	^3^HeMRI V VMAT	FL was segmentedusing a fuzzy c-means clustering algorithm.	** Median reduction: fV_10_ Gy ↓ 1.3%, fV_20_ Gy ↓ 0.8% fMLD Gy ↓ 0.3Gy
Rankine et al. (191)	11	Age NS NSCLC 100% Stage NS	^129^XeMRI V, Gas exchange IMRT(45%) VMAT(55%)	For V and gas exchange maps, a series of isofunction contours were created using thresholds spaced at intervals of 10% relative function.	** Gas exchange-guided FLART demonstrated clinically significant reductions in model-predicted toxicity, more than the accompanying V-guided plans and DVH-based re-optimizations.
Yaremko et al. (192)	27	Age (mean) 69 NSCLC 100% Stage III 93% Stage IV 7%	^3^HeMRI V CRT	Segmentation of FL was performed using semiautomated methods	** Absolute reduction: fV_20_ Gy ↓ 2.4% (AV), ↓ 3.0% (VL), and ↓ 3.5% (NVL) fMLD ↓ 0.8 Gy (AV), ↓ 1.0Gy (VL), and ↓ 1.2 Gy (NVL)

**, denotes statistically significant result; Q, perfusion; V, ventilation; NS, non-specified; FL, functional lung; FLART, functional lung avoidance radiotherapy; AV, anatomic lung; NVL, normally ventilated lung; VL, ventilated lung; gEUfD, generalized equivalent uniform functional dose; fV_x_, functional volume receiving ≥x Gy; V_x_, lung volume receiving ≥x Gy; fMLD, functional mean lung dose; MLD, mean lung dose; RP, radiation pneumonitis; Med, median.

## 5 ViewRay/Unity and Reflexion: new RT technology

### 5.1 MRI-guided RT

RT based on functional imaging aims to preserve lung function by protecting FL regions first. During the treatment process, however, airways reopen due to shrinking of the tumor (207). As tumor anatomy or function changes, adaptive methods are required because the original treatment plan may irradiate lung tissues that were non-functional but are now functional (80). With the development of linear accelerators, MRI-guided linear accelerators (MRLs) with excellent soft tissue resolution have emerged (208–210). MRL can acquire real-time MR images during the treatment process for adjusting treatment plans to accommodate actual positional changes in tumors and normal tissues (211). This MRI-guided RT is called MR-guided RT (MRgRT) and can be used for SBRT of tumors (210). The MRL system includes two main models: ViewRay MRIdian and Elekta Unity. The MRIdian system uses a low magnetic field strength of 0.345 T, while the Unity system uses a conventional magnetic field strength of 1.5 T. Higher magnetic field strength can improve signal-to-noise ratio and imaging quality but may increase geometric distortion compared to low-field systems (210).

#### 5.1.1 ViewRay MRIdian

MRIdian employs 60Co or 6MV linear accelerators and features a distinctive multi-leaf collimator design to improve the accuracy and adaptability of RT (212, 213). The system utilizes tracking and gating techniques to monitor tumor position in real-time, thereby ensuring treatment accuracy. It also optimizes target coverage and minimizes doses to OARs through daily online imaging and plan adjustments (210). A phase I study assessed the feasibility of MR-guided SBRT for super-central lung tumors (214). Results indicated that adaptive re-planning was required in 4 of 10 treatments to achieve target coverage and adhere to OAR dose limits. All five patients in this study attained local disease control within six months, with no occurrences of grade 3 or higher toxic reactions. In comparison to conventional linear accelerators, the MRIdian system offers enhanced visualization of the spinal cord and lumbosacral nerves, resulting in superior dose distribution quality in spinal SBRT (215). Additional studies have documented clinical experiences with MRgRT for both primary and non-primary lung tumors; however, there is a scarcity of clinical outcome reports specifically for NSCLC (216–218). Research indicated that adaptive MRgRT enhances the dose distribution to OARs and improves target coverage relative to non-adaptive methods (219, 220). Additionally, single-fraction image-guided MR-guided SBRT for lung has been documented (221). The integration of single-fraction treatment with real-time image guidance will improve the precision and effectiveness of treatment.

#### 5.1.2 Elekta Unity

In February 2023, Unity received FDA approval for tumor tracking to facilitate ART. The Unity system employs a magnetic field strength of 1.5 T, facilitating multiparametric imaging and the application of diagnostic MRI pulse sequences at this field strength (212). The utilization of high magnetic fields enhances MR image quality, facilitating the reduction of PTV margins, promoting the sparing of OARs, and increasing equivalent toxicity doses (222). Like the MRIdian system, Unity facilitates tracking and gating techniques, enabling plan adjustments via daily online imaging (223). Besides SBRT, the system is capable of performing IMRT, thereby improving treatment accuracy and effectiveness (224). Unity received approval for tumor tracking only last year, resulting in a limited number of studies regarding its application in ART. Prior research has demonstrated the system’s feasibility in spinal SBRT (225). A prospective, multi-institutional, international cohort study registered on clinical trials (NCT04075305) is currently underway to further validate its clinical application. Additional clinical data is anticipated to validate the feasibility of its application in ART planning.

### 5.2 Biology-guided RT (BgRT) - Reflexion X1

Further research on ART indicates that, alongside MRgRT for ART, biology-guided RT (BgRT) is also applicable. The BgRT system utilizes the PET signal from the tumor to direct real-time radiation delivery and can enhance the RT plan according to the tumor’s biological characteristics (225–227). The BgRT mode received FDA approval for patients treatment in February 2023. The Reflexion X1 system represents the inaugural development for BgRT, with its application restricted to the head and neck, chest, abdomen, and pelvic regions (228). The system includes a 6MV linear accelerator, a 16-row kilovolt CT for anatomical positioning, and two megavolt PET detectors for the detection of patient penetrating radiation (229, 230). The X1 machine scans patients after injecting the tracer at the same dosage as the actual treatment to generate PET and CT data needed for treatment planning. PET can processes signals emitted from tumor sites in real-time, allows simultaneous RT for multiple lesions throughout the body (225). Even amid variations in contrast agent and patient movement, the X1 system delivered doses accurately and robustly under static and dynamic conditions (231). Pham et al. (232) demonstrated that the X1 system can produce clinically acceptable IMRT/SBRT plans comparable or superior to Eclipse VMAT. However, the X1 plan requires an extended irradiation duration, necessitating a trade-off between plan quality and efficiency. The standard treatment workflow for BgRT is in development, and a comprehensive process map will assist clinical professionals in the effective, safe, and high-quality implementation of this new technology (233).

### 5.3 Current problems and research direction

The use of MRL in ART may be advantageous based on current research. MRL combined with the previously mentioned MR functional imaging techniques can help facilitate implementation of the FLART plan by providing lung function information. Also, combining FLART with ART plans may allow RT plans to be adjusted in real time based on functional information provided by MR images. Both MRIdian and Unity systems exhibit certain limitations in the treatment of lung cancer. Firstly, lung tissue’s low proton density, differences in magnetization among tissues, and motion-induced artifacts contribute to suboptimal MR imaging outcomes (234, 235). The hardware differences between MR-linear accelerators and conventional diagnostic MRI systems have an impact on image quality and data acquisition (222). Furthermore, MRI does not yield electron density information; therefore, synthetic CT images must be generated using techniques such as volumetric density assignment, atlas-based methods, or artificial intelligence (236–238). The reliability of this method in the chest area is inadequate (238). As compared to MRIdian, Unity’s strong magnetic field influences secondary electron propagation, resulting in a higher dose at the air-tissue interface. Therefore, adjustments to the irradiation field are necessary to mitigate this electron return effect (234, 235). Further, respiratory and cardiac motion can affect the positioning and dose accuracy of tumor targets (239). While both systems can track motion around a target area, only MRIdian can modify irradiation time based on tumor imaging in real-time (222). Moreover, neither system can perform 4D MR imaging, limiting precision in multi-target treatment.

In contrast, BgRT does not require respiratory motion management as tumor motion is relatively static in the 60-RPM (round-per-minute) ring platform (233). The uptake of the PET tracer, primarily fluorodeoxyglucose-FDG, provides functional information regarding the lungs (240). BgRT facilitates ART through real-time monitoring of the PET signal during treatment (231). Functional information derived from PET images can inform BgRT, potentially enabling adaptive FLART plans. Nonetheless, PET-based BgRT technology encounters numerous issues and challenges in clinical applications. Firstly, the spatial resolution of PET is significantly lower than that of anatomical imaging, which does not meet the accuracy requirements of contemporary RT (241). As a result, PET is ineffective at accurately delineating target areas and fails to deliver non-uniform doses to dynamic target regions. Furthermore, BgRT relies on a high tumor signal-to-background noise ratio (TBR); however, FDG uptake is also present in healthy tissues, and TBR may diminish during treatment (231). Thus, cases with inadequate FDG uptake should be treated with conventional CT imaging. Injections of PET tracer and waiting for activity distribution before each treatment extend treatment duration and decrease patient tolerance and RT center efficiency (227). Additionally, medical technicians must have advanced skills and collaborate among various departments during BgRT treatment. Finally, BgRT equipment incurs higher costs due to the inclusion of PET, while the PET tracers used during treatment increase costs further, hindering widespread application. The evaluation of BgRT’s cost-effectiveness and target population should be conducted from a health economics perspective.

## 6 Summary

FLART has been incorporated into clinical trials. The clinical results indicate that FLART effectively reduces HFA dosage, thereby decreasing the incidence of RILI, which supports its clinical feasibility. Nonetheless, numerous practical issues require attention for FLART. Evaluating the suitability of patients for FLART is one aspect of the process. Establishing a comprehensive set of inclusion criteria is essential, encompassing tumor grading, target area size and volume, the relationship and overlap with FL, and underlying lung conditions such as smoking and COPD. Furthermore, different clinical studies have different criteria for classifying FL, and thresholds used in RT plans depend on their own patient cohorts. To be comprehensively applied in clinical practice in the future, guidelines and standardized thresholds must be developed. Presently, the majority of FLART plans predominantly assesses a single lung function imaging modality, either Q or V. Different physiological phenomena, including airway reopening, tumor vessel obstruction, and related thoracic disorders, can result in variations in patient responses to V or Q dosage. In cases of V and Q mismatch, it is advisable to utilize a combination of imaging modalities. However, this patient population is relatively small, as the majority can be sufficiently assessed through either V or Q dosage response alone. The present emphasis of FLART research is primarily on HFA. The literature provides limited information on the assessment of dose volume and functional weight, as strategies to minimize HFA exposure inherently lead to increased LFA dosage. Nonetheless, LFA is not completely non-functional; certain regions may experience temporary loss of functionality, but they may fail to recover once the radiation dose reaches a critical threshold, resulting in irreversible lung damage. Consequently, the development of new technologies is essential for the real-time adjustment of RT planning through the continuous monitoring of OARs, tumor volume, and lung function. The image quality of cone-beam CT from conventional linear accelerators is inadequate for delivering lung function data. The integration of MRI and PET with linear accelerators has enhanced image quality, and research studies indicate the potential of combining FLART with ART. Further clinical research is required to validate its feasibility. Additionally, the DFH index employed in the study solely represents the dose parameters for FL, and reducing the DFH index impacts only the dose in FL, rather than the entire lung. Consequently, a novel functional weighting algorithm is required to enhance both HFA and LFA through the assignment of weights according to functional significance. The DFH derived from the novel weighted optimization algorithm will represent the integrated whole lung dose function, akin to the conventional DVH. The DFH index serves as an intuitive tool for assessing RT planning and forecasting the likelihood of post-radiotherapy toxicity. Further clinical evaluation of new DFH indices, guided by the weighted optimization algorithm, is necessary to establish guidelines and progressively integrate FLART as a routine option for RT.
